# Supplementary material for: BiFC Method Based on Intraorganellar Protein Crowding Detects Oleate-Dependent Peroxisomal Targeting of Pichia pastoris Malate Dehydrogenase
Source: Int J Mol Sci. 2021 May 5;22(9):4890. doi: 10.3390/ijms22094890 (PMC8124512; doi:10.3390/ijms22094890)
Supplement: Supplementary file 1 [file ijms-22-04890-s001.zip › ijms-1165323-supplementary.pdf]

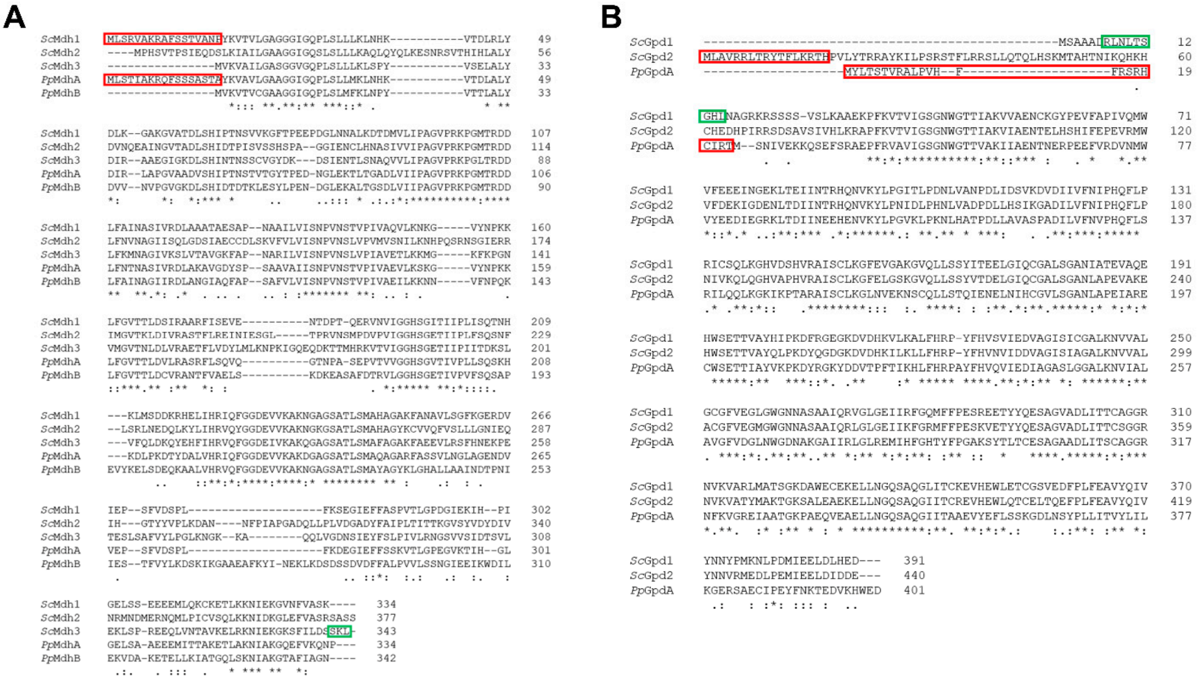

Figure S1. Sequence homologies between dehydrogenases implicated in NADH-shuttling in *P. pastoris* and *S. cerevisiae*. (**A**) Malate dehydrogenases for *S. cerevisiae* (NP_012838.1, NP_014515.2, and NP_010205.1) and *P. pastoris* (XP_002491128.1 and XP_002494265.1). (**B**) Glycerol 3-phosphate dehydrogenases for *S. cerevisiae* (NP_010262.1 and NP_014582.1) and *P. pastoris* (XP_002492095.1). Protein sequences were aligned using the Clustal Omega Multiple Clustal Alignment. * indicates identity and : denotes similarity. Mitochondrial presequences are highlighted in red, and peroxisomal targeting signals are depicted in green.

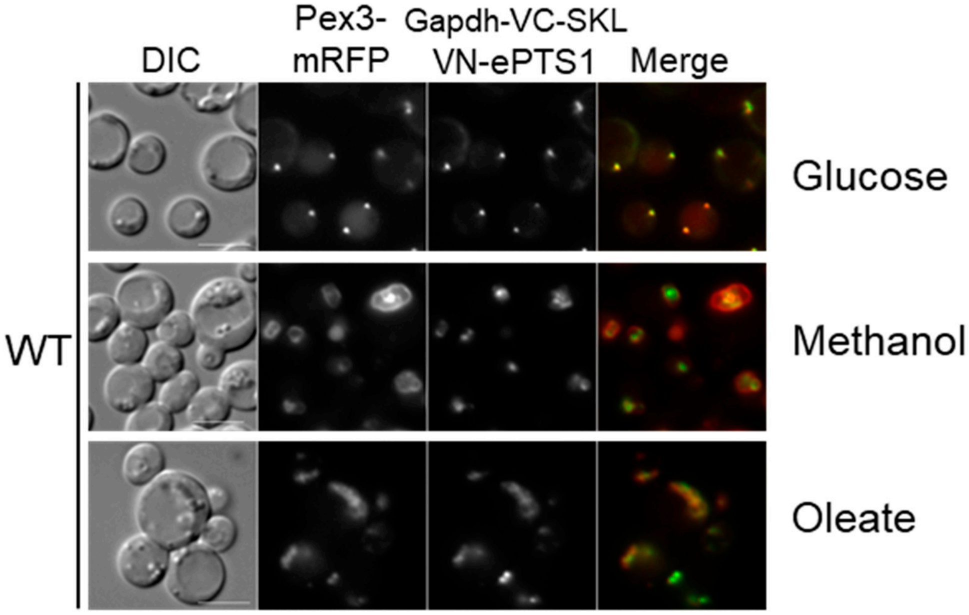

Figure S2. Gapdh-VC with a C-terminal SKL is capable of interaction with VN-ePTS1 at the peroxisome. Strains were grown in glucose, methanol, and oleate overnight. Peroxisomes were visualized using Pex3-mRFP. Bars: 5 μm.

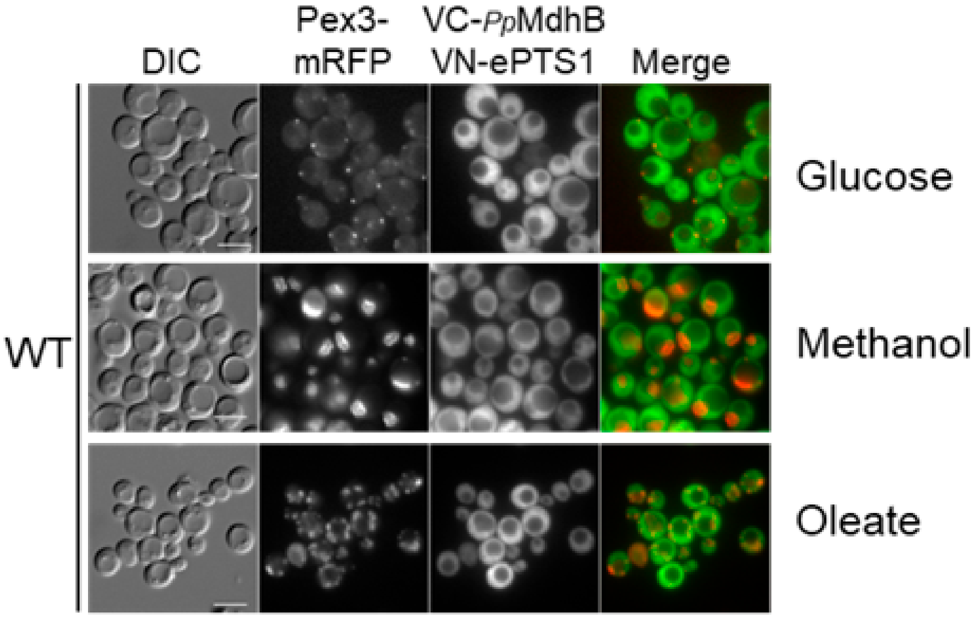

Figure S3. *Pp*MdhB fused with VC at its N-terminus shows only cytosolic localization using the divergent BiFC assay. Strains were grown overnight in glucose, methanol, and oleate. Peroxisomes were visualized using Pex3-mRFP. Bars: 5 μm.

Table S1. Yeast strains.

Yeast Strains	*Description*	*Source*
GS115	*his4*	Lab stock
PPY12h	*his4 arg4*	Lab stock
Δpex7	*Δpex7*::*ARG4 his4*	Lab stock
JC404	*Δpex14*::*ARG4 his4*	Lab stock
sPL66	GS115 + pJCF235-Zeo (P_{PEX3}-*Pex3-mRFP*)::*HIS4*(Zeocin^R) + pPL21 (P_{HTX1}-*Gapdh-VC* + *VN-ePTS1*)::*HIS4*	This study
sPL67	GS115 + pJCF235-Zeo (P_{PEX3}-*Pex3-mRFP*)::*HIS4*(Zeocin^R) + pPL14 (P_{HTX1}-*Pot1-VC* + *VN-ePTS1*)::*HIS4*	This study
sPL68	GS115 + pJCF235-Zeo (P_{PEX3}-*Pex3-mRFP*)::*HIS4*(Zeocin^R) + pPL18 (P_{HTX1}-*MdhA-VC* + *VN-ePTS1*)::*HIS4*	This study
sPL69	GS115 + pJCF235-Zeo (P_{PEX3}-*Pex3-mRFP*)::*HIS4*(Zeocin^R) + pPL19 (P_{HTX1}-*MdhB-VC* + *VN-ePTS1*)::*HIS4*	This study
sPL70	GS115 + pJCF235-Zeo (P_{PEX3}-*Pex3-mRFP*)::*HIS4*(Zeocin^R) + pPL20 (P_{HTX1}-*GpdA-VC* + *VN-ePTS1*)::*HIS4*	This study
sPL75	Δpex7 + pJCF235-Zeo (P_{PEX3}-*Pex3-mRFP*)::*HIS4*(Zeocin^R) + pPL14 (P_{HTX1}-*Pot1-VC* + *VN-ePTS1*)::*HIS4*	This study
sPL77	Δpex7 + pJCF235-Zeo (P_{PEX3}-*Pex3-mRFP*)::*HIS4*(Zeocin^R) + pPL19 (P_{HTX1}-*MdhB-VC* + *VN-ePTS1*)::*HIS4*	This study
sPL79	JC404 + pJCF235-Zeo (P_{PEX3}-*Pex3-mRFP*)::*HIS4*(Zeocin^R) + pPL14 (P_{HTX1}-*Pot1-VC* + *VN-ePTS1*)::*HIS4*	This study
sPL81	JC404 + pJCF235-Zeo (P_{PEX3}-*Pex3-mRFP*)::*HIS4*(Zeocin^R) + pPL19 (P_{HTX1}-*MdhB-VC* + *VN-ePTS1*)::*HIS4*	This study
sPL93	GS115 + pJCF235-Zeo (P_{PEX3}-*Pex3-mRFP*)::*HIS4*(Zeocin^R) + pPL23 (P_{HTX1}-*VC-MdhB* + *VN-ePTS1*)::*HIS4*	This study
sJCF2169	PPY12h + pJCF523(P_{TOM20}-*Tom20-2xmCherry*)::*ARG4*(Hygro^R) pJCF402 *(*P_{GAPDH}-*BFP-SKL)::ARG4 his4*	This study
sJCF2771	sJCF2169 + pPL26(P_{AOX1}-*MdhB-GFP)::HIS4*	This study
sJCF2683	sJCF2169 + pJCF855(P_{GPDA}-*GpdA-GFP)::HIS4*	This study
sJCF2682	sJCF2169 + pJCF854(P_{MDHA}-*MdhA-GFP)::HIS4*	This study
sJCF2770	sJCF2169 + pJCF853(P_{MDHB}-*MdhB-GFP)::HIS4*	This study
sJCF2772	GS115 + pJCF235-Zeo (P_{PEX3}-*Pex3-mRFP*)::*HIS4*(Zeocin^R) + pJCF856 (P_{HTX1}-*Gapdh-VC* + *VN-ePTS1*)::*HIS4*	This study

Table S2. Plasmids.

Plasmid	*Description*	*Source*
pPL14	P_{HTX1}-*Pot1-VC* + *VN-ePTS1, HIS4, AMP*^{R}	This study
pPL18	P_{HTX1}-*MdhA-VC* + *VN-ePTS1, HIS4, AMP*^{R}	This study
pPL19	P_{HTX1}-*MdhB-VC* + *VN-ePTS1, HIS4, AMP*^{R}	This study
pPL20	P_{HTX1}-*GpdA-VC* + *VN-ePTS1, HIS4, AMP*^{R}	This study
pPL21	P_{HTX1}-*Gapdh-VC* + *VN-ePTS1, HIS4, AMP*^{R}	This study
pPL23	P_{HTX1}-*VC-MdhB* + *VN-ePTS1, HIS4, AMP*^{R}	This study
pPL26	P_{AOX1}-*MdhB-GFP, HIS4, AMP*^{R}	This study
pJCF235-ZEO	P_{PEX3}-*Pex3-mRFP, HIS4/Zeocin*^{R}*, AMP*^{R}	Lab stock
pJCF402	P_{GAPDH}-*BFP-SKL, ARG4, AMP*^{R}	Lab stock
pJCF523	P_{TOM20}-*Tom20-2xmCherry, ARG4/Hygro*^{R}*, AMP*^{R}	Lab stock
pJCF853	P_{MDHA}-*MdhA-GFP, HIS4, AMP*^{R}	This study
pJCF854	P_{MDHA}-*MdhA-GFP, HIS4, AMP*^{R}	This study
pJCF855	P_{GPDA}-*GpdA-GFP, HIS4, AMP*^{R}	This study
pJCF856	P_{HTX1}-*Gapdh-VC-SKL* + *VN-ePTS1, HIS4, AMP*^{R}	This study
